# Towards Spectral Variation Analysis: A Data Quality Framework for Non-Targeted Methods

**DOI:** 10.3390/molecules30234597

**Published:** 2025-11-29

**Authors:** Kapil Nichani, Steffen Uhlig, Victor San Martin, Bertrand Colson, Karina Hettwer, Ulrike Steinacker, Heike Kaspar, Petra Gowik, Sabine Kemmlein

**Affiliations:** 1 QuoData GmbH, 01309 Dresden, Germany; 2 Institute of Nutritional Science, University of Potsdam, Arthur-Scheunert-Allee 114-116, 14558 Nuthetal, Germany; 3 QuoData GmbH, 14195 Berlin, Germany; 4 Bundesamt für Verbraucherschutz und Lebensmittelsicherheit, 13347 Berlin, Germany

**Keywords:** non-targeted methods, data quality, MALDI-TOF, MRSA, quality assurance

## Abstract

Non-targeted methods (NTMs) require robust methods for comparing spectral data for reliable classification and identification. Traditional approaches using match factors reduce complex spectral relationships to single values, limiting their utility in quality assurance. This study presents an evaluation of spectral comparison methodologies, contrasting classical Mahalanobis distance (MD) with neural network approaches, namely, neural classification distance (NCD). Using matrix-assisted laser desorption ionization-time of flight (MALDI-TOF) mass spectrometry data from bacterial isolates, we systematically assessed these methods across varying levels of spectral variability. The MD approach exhibited consistent performance under controlled conditions but showed limitations with increasing spectral complexity. In contrast, the NCD demonstrated adaptability across all scenarios, revealing its capability in handling complex spectral relationships. Through this exemplary example, we present the mathematical framework for quantifying spectral variations and establish criteria for method selection in different analytical scenarios. This work provides a foundation for proposing data quality metrics in NTMs and offers practical implementations for routine quality assurance. The methodology developed here extends beyond mass spectrometry applications and contributes to the broader field of analytical quality control in complex spectral analysis.

## 1. Introduction

Non-targeted methods (NTMs) have emerged as a pivotal analytical approach in environmental monitoring [1,2], food safety [3,4], and forensic applications [5,6]. The methodology relies on comprehensive fingerprinting, particularly using measurement data generated by next-generation sequencing, spectroscopy or mass spectrometry, to characterize unknown samples without a priori knowledge of their composition [7]. Sticking to the case of spectral data hereafter, the statistical classification of these spectral patterns into predefined categories (e.g., A or B) can be represented mathematically as:

$f:X\to Y,\;\mathrm{where}\;X\subseteq R^{d}$ represents the d-dimensional spectral data space and Y∈{A,B}.

The reliability of such classifications fundamentally depends on spectral data integrity. Poor quality data inevitably leads to misclassification, with potentially serious consequences in critical applications such as food safety [8] and forensics [9]. Both poor quality training data and “out-of-population” data during inference can equally undermine model performance through subtle degradation mechanisms that often escape standard quality control measures. Despite this criticality, quantitative methods for comparing spectral data remain underdeveloped [10,11,12]. Specifically, the question regarding how to examine variations in spectra arising from (a) time or instrumental factors within single-sample measurements, under repeatability conditions and (b) inter-laboratory differences across multiple samples, under reproducibility conditions. This study attempts to address this gap by proposing new methodologies for spectral data comparison and developing robust metrics for quantifying these spectral variations that will provide a foundation for implementing comprehensive quality assurance protocols in NTM applications. To understand spectral variability in NTMs, we must first consider how measurement error is conceptualized in analytical chemistry.

In traditional analytical measurements, measurement error $\varepsilon_{i}$ for a single measurement point $x_{i}$ is typically characterized as a univariate value $\varepsilon_{i}=x_{i}-\mu_{i}$ and $\varepsilon_{i}∼N\left(0,\sigma_{i}^{2}\right)$ (or some parametric or non-parametric distribution) [13]. Measurement error theory further allows hierarchical decomposition of the error components, e.g., $\sigma_{\mathrm{total}}^{2}=\sigma_{\mathrm{repeatability}}^{2}+\sigma_{\mathrm{laboratory}}^{2}+\sigma_{\mathrm{sample}}^{2}$. However, spectral data presents a more complex scenario, where we have a multivariate measurement. Let us define a spectrum S as an ordered set of d intensity measurements: $S={\{x}_{1},x_{2},\ldots ,x_{d}\}\;\mathrm{where}\;x_{i}\in R$. If μ is a true spectrum, then analogously, ε=S−μ and $\varepsilon ∼N\left(0,\Sigma \right)$, where Σ is the covariance matrix.

The challenge with spectra is already becoming evident here as acquiring a “true” spectrum may be challenging or virtually unattainable. Just like in univariate case, spectral measurement error also exhibits a hierarchical complexity depending on the experimental conditions. At the most fundamental level, variations arise even when spectra are acquired from the same sample, using identical instrumentation, within a single measurement session—representing the inherent instrumental repeatability. The error magnitude typically increases when considering reproducibility: measurements of the same sample across different times, capturing instrument drift and environmental fluctuations. Further complexity emerges in inter-laboratory comparisons, where systematic differences in instrumentation, calibration procedures, and operator practices contribute additional variance components. The highest level of variability manifests when comparing measurements across different sample preparations or batches, incorporating sample heterogeneity and handling effects into the error structure.

The fundamental problem can be formulated as finding a function φ that maps two spectra $S^{1}$ and $S^{2}$ to a set of characteristics C that describe their relationship: $\varphi :\left(S^{1},S^{2}\right)\to C$. The challenge lies in determining φ such that it satisfies: $C=\varphi \left(S^{1},S^{2}\right)$ where $C\in R^{k}$, represents a vector of k characteristics quantifying spectral similarity or dissimilarity. We seek a generalized concept for measurement error for NTMs, and this work is an attempt to propose in this direction.

The hierarchical variations in spectra directly impact method performance characteristics (α), which can be expressed as a function of data quality metrics (Q), sample size (N), and measurement variation (σ): α = g(N, Q, σ). Both Q and σ can be quantified through “distances” or mathematical measures that determine similarity or dissimilarity between spectra. To determine appropriate distance measures for spectral comparison, we must consider both the spectral characteristics (peak positions, shapes, and relative intensities) and the underlying physical phenomena causing variations.

Traditional approaches to spectral comparison face fundamental limitations. Classical statistical methods such as Mahalanobis distance (MD), whilst mathematically rigorous, assume stable covariance structures and become problematic when the number of spectral variables approaches or exceeds the number of observations, the ‘curse of dimensionality’ ubiquitous in spectroscopic data. Conversely, whilst neural network approaches can learn complex spectral relationships, their application to quality assurance in NTM remains largely unexplored. An evaluation comparing these complementary paradigms across varying levels of spectral complexity is notably absent from literature to the best of our knowledge.

**Figure 1:**
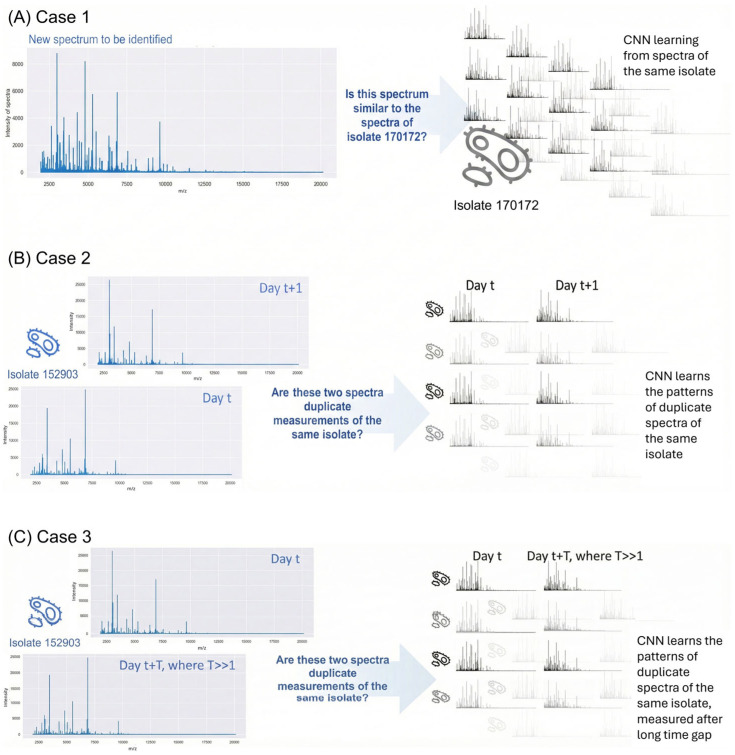
Illustration of 3 cases for the data experiments performed. (**A**) Case 1 for within-run variability. (**B**) Case 2 for day-to-day comparisons, (**C**) Case 3 for cross-run (long time) robustness. Isolate numbers and spectra shown are representative examples.

This study addresses this gap by evaluating MD and neural classification distance (NCD) across hierarchical levels of spectral variability. Rather than positioning these as competing methods, we examine their complementary roles: MD provides a classical statistical framework with clear theoretical underpinnings but inherent dimensionality constraints, whilst NCD offers learned representations that circumvent these limitations but require careful training data curation. We demonstrate these approaches using Matrix-assisted laser desorption/ionisation time-of-flight (MALDI-TOF) mass spectrometry data from Methicillin-resistant *Staphylococcus aureus* (MRSA) and Methicillin-susceptible *Staphylococcus aureus* (MSSA), formulating three cases representing increasing spectral complexity (Figure 1). Each case examines within-type spectral variability (not between MRSA and MSSA). Case 1 examines within-run variability under repeatability conditions (Figure 1A); Case 2 evaluates day-to-day temporal variations (Figure 1B); and Case 3 assesses long-term robustness across three-month intervals (Figure 1C).

Through these exemplary cases, we establish criteria for method selection in different analytical scenarios, identify fundamental limitations of classical approaches, and demonstrate how NCD addresses these constraints. This work provides a basis for implementing comprehensive quality assurance protocols in NTM applications.

## 2. Results

We first evaluated MD performance across the three experimental scenarios to establish a classical statistical baseline. Subsequently, we assessed the NCD approach to examine whether learned representations could address the limitations observed with MD.

### 2.1. Results for MD for Different Scenarios of Spectral Variability

**Figure 2:**
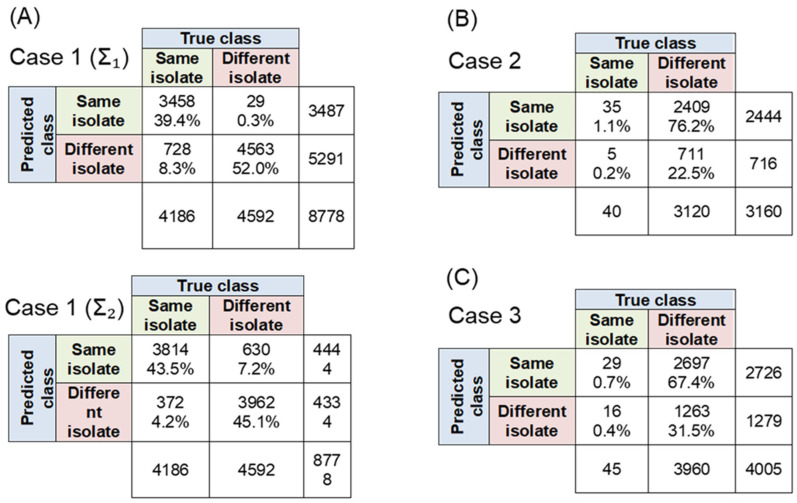
Confusion matrices for the three cases for calculation of Mahalanobis distances: (**A**) case 1, (**B**) case 2 and (**C**) case 3.

Figure 2 shows the results for MD evaluated for the three cases. In Case 1 (Figure 2A), we evaluated MD using a dataset of 92 spectra from one MSSA isolate (class 1, same isolate) and 41 spectra from different MSSA isolates (class 2, different isolates), yielding 8778 pairwise comparisons. We tested two covariance matrix approaches: Σ_1_ derived from 60 same-isolate measurements and Σ_2_ derived from 30 different-isolate measurements.

Using Σ_1_, the expected MD between corresponding spectra was empirically set to 12, rather than using the theoretical value of $\sqrt{\left(2\times 60\right)}=10.95$. Since raw distance values alone were insufficient for isolate classification, we established a decision threshold to categorize samples into class 1 (same isolate) or class 2 (different isolate). The threshold of 13.77 (*p* = 0.95) was determined using an empirical expected MD value of 12 multiplied with the inverse chi-squared distribution. This was calculated as (12 × √(CHISQ.INV (0.95, df = 60)/60)), where CHISQ.INV (0.95, 60) = 79.08. MD achieved 91.4% efficiency (8021 correctly classified pairs: 4563 true negatives and 3458 true positives), with 8.3% false positives and 0.3% false negatives.

The alternative approach using Σ_2_ with a threshold of 7.0 yielded similar performance at 88.6% efficiency (7776 correctly classified pairs: 3814 true positives and 3962 true negatives), with 4.2% false positives and 7.2% false negatives. These results demonstrate that MD-based classification is feasible under repeatability conditions, though not without classification errors.

Moving to Case 2 (Figure 2B) using 40 pairs of MRSA spectra on a “day t” were split into groups of 30 and 10 spectra, respectively. The former is used for the calculation of Σ. Remaining 10 spectra from class 2, are set aside and not used in the calculation of Σ. Additional 40 spectra of the same isolates measured on day t + 1 from the same isolates were used in the pairwise distance calculations.

MD analysis using the day-t covariance matrix achieved very few correct classifications (35 + 711 = 746 out of 3160) with a 9.35 threshold. The large increase in false positive rate (76.2%) compared to the previous scenario suggests limitations in MD’s ability to capture complex spectral relationships.

Lastly, in Case 3 (Figure 2C), using 45 pairs of MSSA spectra with maximum expected variation, MD correct classifications remained poor (29 + 1263 = 1292 out of 4005), with a 67.4% false positive rate. This significant decrease in performance indicates MD’s limitations in handling highly variable spectral relationships.

The progressive decline in MD performance across the three cases reveals fundamental limitations of this classical approach. When the number of spectral variables approaches or exceeds the number of observations, the sample covariance matrix becomes ill-conditioned or singular, compromising MD calculation. Moreover, MD assumes a static covariance structure (**Σ**), which may not capture temporal variations or complex non-linear spectral relationships. The ‘curse of dimensionality’ is particularly acute in spectroscopic data where the number of *m*/*z* channels typically far exceeds the number of spectra.

These inherent constraints necessitate alternative approaches that can remain stable in high-dimensional spaces and adapt to varying spectral complexity. NCD offers such an alternative, circumventing dimensionality constraints through learned feature representations rather than explicit covariance estimation.

### 2.2. Results for NCD for Different Scenarios of Spectral Variability

**Figure 3:**
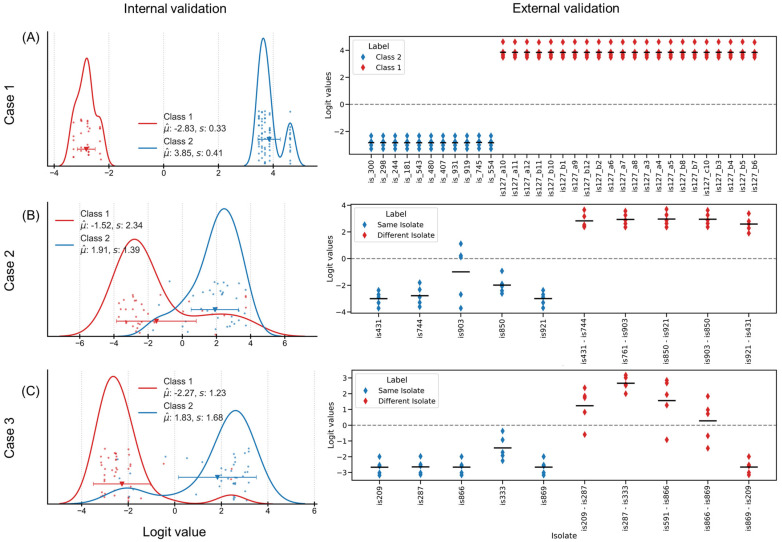
Summary of NCD results for the three cases: (**A**) case 1, (**B**) case 2 and (**C**) case 3. Left panels show distribution of logit values for internal validation. The right panels show the distribution for external validation samples.

In Case 1, a CNN was trained with 5-fold nested cross validation (NCV) for classifying spectra corresponding to class 1 (same isolate) and class 2 (different isolates). The external validation set comprised 22 spectra from class 1 and 11 spectra from class 2. The NCD demonstrated superior discrimination capability, achieving 100% classification efficiency in both internal (*n* = 70) and external (*n* = 33) validation set (considering zero as decision threshold) (Figure 3A). The clear separation in NCD indicates robust feature extraction beyond linear distance. Absence of misclassifications establishes NCD as a reliable distance value for discriminating spectra under repeatability conditions, providing a good basis for more complex temporal comparisons.

Turning to Case 2, here the differences in the spectra were used to construct a set of data for the training a CNN. On one hand, the difference between the replicates (measured a day apart) were used to generate a collection of forty samples that each represented data from the same isolate. On the other hand, for the “counter class”, spectra from randomly selected isolates were subtracted, resulting in the creation of another dataset containing forty samples. For the purposes of training and verifying the model, only 70 of the initial 80 samples were utilized. They were divided into five equal folds, each containing a total of 14 samples. The remaining 10 samples were utilized for the purpose of conducting an external validation. Figure 3B shows the logit values for the external validation dataset. All spectra of the classes 1 (difference between replicate of the same isolate) and 2 (difference between two different isolate) were classified correctly. The CNN models, trained on spectral differences, maintained stronger performance with 81% of the spectra correctly classified in internal validation (*n* = 70) and 100% in external validation (*n* = 10). One isolate “is903” with its large distribution of the logit values, close to the decision threshold, appears to be a “challenging sample”.

Lastly, for Case 3, another CNN was trained similar to the one applied for the previous case where class 1 comprised 45 samples (differences between spectra of the same isolate measured in two different rounds) and class 2 comprised 45 samples (differences between spectra from two isolates measured during the same round). 10 samples were separated for external validation, and the remaining 80 samples were organized in 5 folds of 16 samples each. Figure 3C shows the distribution of NCD, with 90% efficiency in internal validation (*n* = 80) and 80% in external validation (*n* = 10). The logit value distribution reveals-maintained class separation, though with reduced margin compared to simpler scenarios, indicating systematic handling of complex spectral relationships.

Overall, these results demonstrate that while both distances (MD and NCD) can effectively discriminate spectral differences under controlled conditions, NCD maintains more robust performance as spectral complexity increases. The progressive decline in MD performance contrasted with the relatively stable CNN efficiency suggests fundamental differences in how these methods handle spectral variation.

Overall, the comparative performance of MD and NCD across the three experimental scenarios reveals markedly divergent directions. In Case 1, both approaches achieved strong discrimination under repeatability conditions, with MD attaining approximately 90% efficiency and NCD achieving 100% efficiency in both internal and external validation. However, as spectral complexity increased through temporal variations, performance diverged substantially. In Case 2, MD efficiency declined sharply to 23.6% (746 correct out of 3160 pairs), whilst NCD maintained 81–100% efficiency across internal and external validation sets. This pattern continued in Case 3, where MD achieved only 32.3% efficiency (1292 correct out of 4005 pairs) under long-term variability, compared to NCD’s 80–90% efficiency. The systematic degradation of MD performance, from 91.4% under controlled conditions to below 33% under temporal variability, contrasts sharply with NCD’s relatively stable performance across all scenarios. These results demonstrate that whilst both distance metrics can effectively discriminate spectral differences under controlled conditions, NCD maintains robust performance as spectral complexity increases, suggesting fundamental differences in how these methods handle spectral variation beyond simple repeatability conditions.

## 3. Discussion

This study evaluated two complementary paradigms for quantifying spectral variations in NTM: classical statistical distances and learned neural representations. Our findings establish that method selection should be guided by inherent limitations rather than universal superiority claims. MD provides a mathematically rigorous framework with clear theoretical underpinnings, making it suitable for controlled scenarios where covariance structures are stable and dimensionality constraints are manageable. However, its performance degrades systematically with increasing spectral complexity, declining from >90% efficiency under repeatability conditions (Case 1) to <25% efficiency under long-term variability (Case 3). This degradation is not a failure of the method per se but rather reflects its fundamental assumptions: stable covariance matrices and adequate observation-to-variable ratios.

The MD performance degradation is mathematically inevitable given the dimensionality structure: with q ≈ 20,000 *m*/*z* channels and *n* ≤ 80 observations, the sample covariance matrix rank is bound by *n*, rendering Σ^^^ singular or near-singular. This is not a failure of MD as a distance metric, but rather a fundamental incompatibility between classical covariance-based approaches and the high-dimensional, low-sample-size (HDLSS) regime characteristic of spectral data.

NCD addresses these specific limitations. By learning feature representations directly from data, it circumvents explicit covariance estimation and dimensionality constraints. The maintained performance across all scenarios (80–100% efficiency) demonstrates its adaptability to varying spectral complexity. Crucially, NCD’s learned embedding function φ adapts to temporal changes, capturing subtle spectral variations that violate the static covariance assumption underlying MD. This adaptability comes at the cost of requiring carefully curated training data and lacking the transparent theoretical framework of classical statistics.

The choice between these approaches depends on the analytical context. For within-run quality control where repeatability is expected, MD offers computational simplicity and theoretical clarity. For applications involving temporal variations, cross-laboratory comparisons, or high-dimensional data, NCD provides necessary robustness. In practice, both methods can serve complementary roles: MD for initial method validation under controlled conditions, and NCD for ongoing quality assurance in operational settings.

These findings directly address the fundamental question posed in the introduction: how to quantify spectral variations arising from (a) instrumental factors under repeatability conditions and (b) inter-laboratory differences under reproducibility conditions. The hierarchical structure of our three cases—progressing from within-run to day-to-day to long-term variability—demonstrates that distance metrics must scale with experimental complexity. The transition from single-value match factors to multidimensional distance measures (whether MD or NCD) represents a significant advancement, but our results show that dimensionality-robust approaches are essential for operational NTM.

Several limitations warrant consideration. First, this study employed a single analytical platform (MALDI-TOF MS) with specific bacterial types. Whilst the mathematical frameworks are technique-agnostic, empirical validation across diverse spectral modalities (infrared, Raman, nuclear magnetic resonance) remains necessary. Second, the NCD approach requires carefully curated training data representing relevant spectral variations. In scenarios where such training data are unavailable or impractical to generate, classical statistical approaches may remain preferable despite their limitations. Third, computational requirements differ substantially: MD calculation is immediate, given a covariance matrix, whilst NCD requires model training. For real-time applications, this computational overhead may influence method selection. Finally, the interpretability of learned neural representations remains less transparent than classical statistical distances, potentially limiting regulatory acceptance in some domains.

The integration of these distance metrics into quality assurance (QA) frameworks realizes the vision outlined in the introduction: establishing quantitative criteria for spectral data integrity. Unlike traditional match factors that reduce spectral complexity to single similarity values, obscuring multidimensional variations and failing to account for measurement conditions, both MD and NCD provide hierarchical distance measures that can capture repeatability and reproducibility variations. The quantitative logit values (NCD) or distance values (MD) serve dual purposes for both performance evaluation and data quality assessment [7]. They provide numerical and graphical interpretation of spectral similarity whilst offering objective thresholds for classification decisions. This objectivity enables several practical applications for routine quality assurance in NTM.

First, the integration of NCD with traditional control charts demonstrates particular promise, offering a robust framework for continuous quality monitoring that extends beyond conventional targeted approaches. The application of control charts introduces a systematic approach previously difficult to implement in non-targeted analysis. By monitoring NCD over time, laboratories can now detect subtle deviations in spectral patterns that might indicate instrumental drift or sample preparation inconsistencies. This capability is particularly valuable given the complex nature of NTMs, where traditional quality control parameters may be insufficient or inappropriate.

Second, the distances provide quantitative outlier identification as well. By leveraging the distances between spectra, the method provides a quantitative basis for identifying anomalous measurements. This objective approach to outlier detection strengthens the reliability of spectral databases and supports more confident data interpretation in NTMs.

Third, NCD offers objective means for evaluating spectral preprocessing strategies, enabling laboratories to optimize or standardize their analytical workflows based on quantitative evidence rather than empirical observation.

Perhaps most significantly, this work establishes a foundation for new proficiency testing schemes specifically designed for NTM. The use of NCD offers a novel approach to inter-laboratory comparisons, potentially enabling standardized quality assessment across different laboratories and instruments. This development addresses a long-standing challenge in the field of non-targeted analysis, where traditional proficiency testing approaches have proven inadequate.

These findings extend beyond MALDI-TOF MS to the broader field of non-targeted methods, where similar challenges in spectral comparison and quality assurance persist. The NCD approach provides a universal framework for developing robust quality metrics across analytical techniques using spectral data. These advancements collectively suggest a path toward more robust and reliable NTMs, with implications for fields ranging from environmental monitoring to food safety analysis. Future work should focus on the standardization of these approaches and their integration into routine laboratory practice.

## 4. Materials and Methods

### 4.1. Bacterial Isolates and MALDI-TOF Data Acquisition

Bacterial cultures were prepared on Columbia sheep blood agar plates and incubated aerobically at 33–37 °C for 18–24 h. Sample preparation followed the direct transfer method [14,15], bacterial colony material was transferred directly onto steel MALDI target plates and overlaid with 1 µL of α-cyano-4-hydroxycinnamic acid (HCCA) matrix solution, then air-dried at room temperature.

Mass spectra were acquired using a Bruker MALDI-TOF mass spectrometer operating in positive linear mode [15,16]. The instrument employed a nitrogen UV laser (337 nm) at 20 Hz frequency. Acceleration voltages were 20.0 kV (ion source 1) and 18.5 kV (ion source 2), with pulsed ion extraction of approximately 250 ns [17]. Spectra were recorded in the mass range of 2000–20,000 Da, typical for bacterial protein profiling [15,16,17]. Each spectrum represented the sum of approximately 240 laser shots using the manufacturer’s AutoX acquisition method [18]. External calibration was performed using Bruker Bacterial Test Standard (*Escherichia coli* extract) according to manufacturer protocols [19]. Raw spectral data were exported for subsequent processing and analysis.

### 4.2. MALDI-ToF Spectra Processing

The spectra underwent baseline correction removed background noise by estimating (rolling median) and subtracting a baseline from each spectrum. The available dataset of 923 MALDI-TOF mass spectrometry measurements, 658 MSSA and 265 MRSA, were used. This dataset fortuitously provided measurements across different time points and experimental runs, enabling construction of three distinct analytical scenarios representing varying levels of spectral variability. The data compilation was done by looking at the isolate numbers, type of isolate, and whether it was a repeat measurement.

### 4.3. Within-Run and Across-Run Variability

We systematically constructed three test cases that would build an understanding of spectral relationships. By strategically partitioning the available measurements, we created complementary scenarios progressing from fundamental within-run variability (Case 1) through temporal comparisons (Case 2) to cross-run (long time) robustness (Case 3).

Case 1 investigates whether spectral patterns can distinguish repeat measurements of a single isolate from different isolates. The dataset comprises 92 same-day spectra from one MSSA isolate and 92 spectra from 41 different MSSA isolates. This allows evaluation of same-day measurement detection capability.

Case 2 explores more subtle spectral differences, specifically whether spectra from the same isolate measured one day apart remain distinguishable from different isolates measured simultaneously. The dataset contains 40 MRSA isolates, each measured twice one-day apart.

Case 3 examines long-term spectral variability using 45 MSSA isolates measured twice, approximately three months apart. The analysis determines whether same-isolate spectra maintain closer relationships than different-isolate spectra measured on the same day, despite the extended time gap.

For each case, we evaluated the spectral relationships using NCD and MD. NCD was derived by training convolutional neural network (CNN) models to distinguish between within-isolate and between-isolate spectral differences, offering a machine learning perspective on spectral discrimination. This dual approach strengthened our assessment of spectral relationships across increasing levels of experimental variability.

### 4.4. Mahalanobis Distance (MD)

We examined spectral differences through classical multivariate statistical lens using the Mahalanobis distance (MD). Consider a set of *n* reference spectra represented by the data matrix $X\in R^{n\times q}$, where each row represents a spectrum $X_{i}\in R^{q},i=1,\ldots ,n$. For mass spectrometry data, q represents the number of mass-to-charge (*m*/*z*) channels, and each element $x_{ij}$ represents the intensity at the jth *m*/*z* value in spectrum i. The Mahalanobis distance $MD\left(X_{i}\right)$ between a spectrum $X_{i}$ and the distribution of reference spectra is defined as:

(1) $$MD\left(X_{i}\right)=\sqrt{{\left(X_{i}-\mu \right)}^{⊤}\Sigma^{-1}\left(X_{i}-\mu \right)}$$

where $\mu \in R^{q}$ is the mean spectrum of the reference set and $\Sigma \in R^{q\times q}$ is the covariance matrix (Equation (2)), which can be calculated as:

(2) $$\Sigma =\frac{1}{n-1}{\left(X-\mu \right)}^{⊤}\left(X-\mu \right)$$

The covariance matrix describes the correlations between multiple variables. It is a square matrix with dimensions equal to the number of variables, and its entries represent the covariances between the variables. The diagonal entries of the covariance matrix represent the variances of the individual variables, which measure the dispersion or spread of the data around the mean. The off-diagonal entries represent the covariances between pairs of variables, which measure the extent to which the variables are linearly related. The expected value of the Mahalanobis distance $E\left(MD\right)$ for d degrees of freedom can be derived as $\sqrt{2d}$.

### 4.5. Neural Classification Distance (NCD)

Alongside MD, in this study, we introduce the Neural Classification Distance (NCD), which quantifies spectral variations through neural network-based classification probability. A CNN was employed to learn discriminative features directly from spectral data; however, the specific architectural details are not the focus of this work. Rather, we emphasise that the CNN serves as a tool to generate a learned embedding of spectral relationships, which is then transformed into an interpretable distance metric [20].

The CNN models were implemented using TensorFlow 2 with Keras API in Python 3.9. Representative configurations consisted of 2–3 one-dimensional convolutional layers (4–8 filters per layer, kernel sizes 8–32) designed to capture local spectral patterns, followed by max-pooling layers (pool size 4) for dimensionality reduction. Dropout regularization (rate 0.3) and L2 weight regularization (λ = 0.001) were employed to prevent overfitting [21]. The convolutional layers were followed by flattening, a dense layer (64–128 units), and sigmoid output activation for binary classification. Models were compiled with the Adam optimizer (learning rate 0.0001–0.001) and binary cross-entropy loss function. The architecture was selected to provide adequate classification performance whilst avoiding overfitting through nested cross-validation.

Mathematically, let $f_{\theta }:R^{q}\to \left[0,1\right]$ be a neural network with parameters θ that maps input spectra to classification probabilities $\left(p\right)$. This network function can be decomposed as: $f_{\theta }=g\circ \phi$, where $\phi :R^{q}\to R^{k}$ is the internal transformation through hidden layers and $g:R^{k}\to \left[0,1\right]$ is the final sigmoid activation function. The internal transformation ϕ implements a non-linear mapping through M hidden layers:

(3) $$\phi \left(X_{i}\right)=W_{M}\sigma \left(W_{M-1}\sigma \left(\ldots \sigma \left(W_{1}X_{i}+b_{1}\right)\ldots \right)+b_{M-1}\right)+b_{M}$$

where $W_{M}$ are the weight matrices, $b_{M}$ are bias vectors and $\sigma \left(\cdot \right)$ is a non-linear activation function. In our implementations, activation functions included LeakyReLU or Scaled Exponential Linear Units (SELUs) to prevent vanishing gradients. The NCD is then defined through the logit transformation (Equation (4)):

(4) $$NCD\left(X_{i}\right)=l\left(f_{\theta }\left(X_{i}\right)\right)=\log\left(\frac{p}{1-p}\right)$$

where $p=f_{\theta }\left(X_{i}\right)$ is the network’s output probability for spectrum i and $l\left(\cdot \right)$ is the logit transformation.

Neural network classifiers were trained to distinguish between spectral classes, incorporating both repeatability and reproducibility aspects. These logit-transformed probabilities serve as a natural distance metric, with values further from zero indicating greater distinction between classes. This distribution inherently captures prediction uncertainty, encompassing both analytical and spectral variations. The NCD offers several advantages: (i) maps high-dimensional spectra to an interpretable scalar value, (ii) captures non-linear relationships between spectral features, (iii) provides an unbounded measure that effectively captures subtle variations in spectral quality, (iv) learns relevant patterns from training data.

To ensure robust model selection and unbiased performance estimation, NCV is recommended. This involves splitting the dataset into a calibration set for training and finetuning, and an external validation set for independent assessment. The calibration set undergoes k-fold cross-validation, yielding k different models that collectively assess performance stability. This approach prevents overfitting and yields reliable estimates of the model’s generalization ability. Critically, the focus of this work is not on optimizing neural network architecture but rather on establishing NCD as a robust distance metric for spectral quality assessment. Alternative architecture could equally serve this purpose provided they achieve adequate classification performance.

### 4.6. Performance Evaluation

The performance of MD approaches was evaluated using confusion matrices, which tabulate the agreement between predicted classifications and true class labels. For binary classification of spectral pairs into “same isolate” (class 1) or “different isolate” (class 2), the confusion matrix comprises four elements: true positives (TPs), correctly classified same-isolate pairs; true negatives (TNs), correctly classified different-isolate pairs; false positives (FPs), different-isolate pairs incorrectly classified as same isolate; and false negatives (FNs), same-isolate pairs incorrectly classified as different isolate.

Classification efficiency (Equation (5)), often referred to as accuracy in ML literature, is a metric representing the proportion of correctly classified spectral pairs relative to the total number of pairs evaluated was calculated as:

(5) $$Efficiency\;=\frac{TP+TN}{TP+TN+FP+FN}\;\times 100\%$$

For MD, classification was performed by comparing calculated distances against empirically determined thresholds. For NCD, the distributions were directly plotted to visualize class separation, with zero as decision boundary, with negative values indicating same-isolate classification and positive values indicating different-isolate classification.

## 5. Conclusions

This study establishes that spectral comparison methodology in NTM must account for both the strengths and inherent limitations of available approaches. Classical MD provides acceptable performance under controlled repeatability conditions (>90% efficiency, Case 1) but faces fundamental constraints in high-dimensional settings and under temporal variability, achieving only 24% efficiency in long-term comparisons (Case 3). These limitations arise from dimensionality constraints and static covariance assumptions, not from methodological inadequacy. NCD addresses these specific limitations, maintaining 80–100% efficiency across all scenarios through learned representations that adapt to spectral complexity.

Beyond methodological evaluation, this work establishes practical frameworks for implementing data quality metrics in routine analytical laboratories. The distance-based approaches enable: (i) automated outlier detection through quantitative thresholds; (ii) objective evaluation of spectral preprocessing strategies; (iii) continuous quality monitoring through control charts; and (iv) standardized inter-laboratory proficiency testing schemes specifically designed for NTM. These capabilities address long-standing challenges in non-targeted analysis where traditional quality control parameters are insufficient. Whilst demonstrated using MALDI-TOF mass spectrometry, the principles extend to other spectral techniques in environmental monitoring, food safety, and forensic applications. The mathematical frameworks presented (Equations (1)–(4)) are technique-agnostic, requiring only appropriate adaptation of preprocessing and feature extraction. Future research should validate these approaches across diverse analytical platforms and establish community-wide standards for quality assessment in NTM. The limitations identified here, particularly regarding training data requirements for NCD and dimensionality constraints for MD, warrant further investigation to define operational boundaries for method selection.

## Data Availability

Data is available on request because the data are subject to restrictions of a multi-year monitoring effort.

## Abbreviations

The following abbreviations are used in this manuscript:

CNN	Convolutional neural network
*m*/*z*	Mass-to-charge ratio
MALDI-TOF	Matrix-assisted laser desorption/ionization time-of-flight
MD	Mahalanobis distance
MRSA	Methicillin-resistant Staphylococcus aureus
MSSA	Methicillin-susceptible Staphylococcus aureus
MS	Mass spectrometry
NCD	Neural classification distance
NCV	Nested cross-validation
NTM	Non-targeted methods
QA/QC	Quality assurance/quality control
